# Handgrip strength as a predictor of one-year mortality in elderly patients with fragility hip fracture

**DOI:** 10.1007/s40520-025-03019-2

**Published:** 2025-04-02

**Authors:** Francesco Salis, Irene Buffoli, Maristella Belfiori, Alice Bellisai, Benedetta Gianoglio, Giuseppe Marongiu, Monia Marzuolo, Giuseppe Navarra, Veronica Piras, Benedetta Puxeddu, Luisa Sanna, Chiara Scudu, Antonio Capone, Antonella Mandas

**Affiliations:** 1 Department of Medical Sciences and Public Health, University of Cagliari, SS 554 Bivio Sestu, Monserrato, Cagliari, 09042 Italy; 2 Department of Biomedical Sciences, University of Cagliari, Cagliari, Italy; 3 Department of Surgical Sciences, University of Cagliari, Cagliari, Italy; 4 University Hospital “Azienda Ospedaliero-Universitaria” of Cagliari, Monserrato, Cagliari, Italy

**Keywords:** Handgrip strength, Fragility fractures, Elderly, Mortality

## Abstract

**Background:**

Fragility fractures occur on porotic bones due to minor trauma and are associated with high rates of disability and mortality.

**Aims:**

To evaluate the ability of handgrip strength to predict one-year mortality in elderly patients with fragility hip fracture.

**Methods:**

We enrolled patients aged 65 years and older with fragility hip fractures admitted to an Italian orthopedic unit. They underwent a comprehensive geriatric assessment, including handgrip strength measurement, and all received surgical intervention.

**Results:**

Among the 322 enrolled patients (median age: 84 years; 75.2% women), the one-year mortality rate was 15.5%. According to the European Working Group on Sarcopenia in Older People 2 guidelines, 235 subjects (73.0%) exhibited low handgrip strength. This group revealed HR: 2.36 (95%CI: 1.06–5.24) for one-year mortality compared to the group with adequate handgrip strength (*p* = 0.036). After adjusting for age and risk of adverse event, through Multidimensional Prognostic Index, the HR decreased to 1.31 (95%CI: 0.56–3.07), with a lower validity.

**Discussion:**

Our study found a slightly lower one-year mortality than other studies with similar samples, probably due to the co-management of orthopedic and geriatric teams. As for the main outcome, low handgrip strength was significantly associated with one-year mortality. However, the significance diminished when considering possible confounding variables, despite a lower precision of the model.

**Conclusions:**

Low handgrip strength predicts one-year mortality in elderly people with fragility hip fractures. Further studies are needed to explore the possible influence of confounders.

## Introduction

Fragility fractures represent one of the major public health problems in the elderly population, since they are usually associated with serious complications, such as complex disability, loss of independence, reduced quality of life and increased mortality [1, 2]. These fractures occur on porotic bones, following minor trauma [3]. They can affect all skeletal segments, and the vertebral body, the proximal epiphysis of femur and humerus, and the distal epiphysis of the radius are usually involved [3]. The epidemiological impact of fragility fractures is significant: literature estimates suggest that up to 9% of elderly individuals die within a month of the event and up to 36% within a year [1, 3, 4]. Fragility fractures imply osteoporosis, and when it is combined with sarcopenia, understood as a reduction in muscle strength, quality, and quantity of muscle mass, and reduced physical performance, the risk of subsequent disability increases significantly [5]. Also, muscular weakness increases the risk of falls and fracture itself [6, 7]. The biochemical and clinical bond between muscle and bone has led to the coinage of the term “osteosarcopenia”, which identifies a geriatric syndrome increasing the risk of negative outcomes in the elderly population [8–11].

Various studies have investigated the predictive factors for mortality in the elderly with fragility fractures, identifying advanced age, frailty, comorbidities, reduced pre-fracture mobility, delayed surgical intervention, cognitive decline, malnutrition, and reduced muscle strength, as key determinants [5, 12–17]. As for muscle strength, it goes through three main stages during life: it increases to a peak in early adulthood, then remains stable during middle age, to progressively decline thereafter. This decline is particularly evident in the elderly, affecting around one out of four people [18, 19]. In this regard, a prospective cohort study conducted in a Korean population demonstrated that muscle weakness was strongly associated with an increased risk of all-cause 10-year mortality [20].

Among the various diagnostic tools for assessing muscle strength, the handgrip strength test has proven to be one of the simplest, quickest, and most reliable methods [21, 22]. It can be accurately measured using a portable dynamometer, being more convenient and less expensive than other methods. The PURE study, that involved over 140,000 participants from 17 countries, showed that for every 5 kg reduction in handgrip strength, the risk of mortality increased by 16% [23]. A meta-analysis conducted in 2018 further confirmed the importance of handgrip strength as a predictor of mortality, consolidating the results of multiple investigations showing that individuals with reduced grip strength have a higher risk of dying from any cause, regardless of age or physical activity levels [24].

Also, its measurement has been widely used to identify sarcopenia, with specific thresholds established by the European Working Group on Sarcopenia in Older People 2 (EWGSOP2) guidelines: a handgrip strength of less than 27 kg in men and less than 16 kg in women is suggestive for sarcopenia [25, 26], for the diagnosis of which it is also necessary to perform an analysis of muscle quantity [25]. However, grip strength as an independent factor has received much less attention, and uncertainties remain regarding the clinical interpretation of individual grip strength measurements as an independent risk factor for predicting mortality in elderly patients with hip fractures [27].

For these reasons, the aim of this study is to evaluate the ability of handgrip strength to predict one-year mortality in elderly patients with fragility hip fracture.

## Methods

The primary outcome of the investigation was the evaluation of one-year mortality. Hence, it took the form of a prospective study, encompassing individuals admitted to the Orthopedic and Traumatology Unit of the University Hospital of Monserrato, Cagliari, Italy, from November 2021 to September 2023 for fragility hip fracture. Exclusion criteria included: (1) individuals under the 65 years of age, (2) in a coma or with hypokinetic delirium, (3) with a concurrent condition that would prevent the assessment of handgrip strength (e.g., concurrent fracture of the upper limb, recent hand surgery, severe peripheral neuropathy), and (4) who did not provide informed consent.

In consideration of a 95% confidence level, a 5% confidence interval, an anticipated prevalence (P) of 0.5, a Z-score (z) of 1.96, and an error margin (e) of 6%, the requisite sample size (N) was calculated to be at least 267 subjects, as calculated by the formula:

$$\:N=\:\frac{{z}^{2}*P\:(1-P)}{{e}^{2}}$$

### Assessment

The enrolled subjects underwent a comprehensive geriatric assessment (CGA), the assessment of handgrip strength, and the collection of a sample of venous blood, upon admission.

#### Comprehensive geriatric assessment

The CGA was performed with validated tools administered by physicians with specific training in geriatrics. It included the assessment of cognitive capacities (Short Portable Mental Status Questionnaire [28]), mood (Geriatric Depression Scale [29]), nutritional status (Mini Nutritional Assessment [30]), risk of pressure ulcers (Exton-Smith scale [31]), and the extent of comorbidities (Comorbidity Index Rating Scale [32]) upon admission. It also included the assessment of the independence in basic and instrumental activities of daily living (BADL and IADL [33]), referred to the moment prior to the trauma that led to the hip fracture. The above reported scores, together with the number of medications taken, and social status, were used to calculate the Multidimensional Prognostic Index (MPI) [34], which is a tool validated to predict the risk of adverse event in elderly people. In detail, the scores of the abovementioned tools are divided into three categories with decreasing limitations, and each category is assigned a score of 0, 0.5, or 1, respectively. The average of the sum of the scores will therefore be a number between 0 and 1. Scores below 0.33 suggest a low risk of adverse events, scores of at least 0.66 suggest a high risk, while intermediate risk is indicated by scores between 0.33 and 0.66. This tool is useful to stratify elderly patients in various clinical settings [35–38]. Information on the clinical history of previous fractures was also collected.

#### Assessment of handgrip strength

Handgrip strength was measured using a mechanical dynamometer. Two tests were performed for each upper limb, and the best result was recorded. According to the EWGSOP2 guidelines [25], the cut-off values used to distinguish between adequate and reduced muscle strength were 27 kg for males and 16 kg for females.

#### Venous blood sample

The venous blood sample was collected upon admission to the Orthopedic and Traumatology Unit to measure: hemoglobin, serum creatinine, cholinesterase, albumin, vitamin B9, vitamin B12, vitamin D, parathormone, thyroid stimulating hormone, and C-reactive protein.

All the enrolled patients subsequently underwent orthopedic surgery, and orthopedic and geriatric follow-up for one year.

### Statistical analysis

The Shapiro-Wilk test was used to assess normal distribution of the variables. The variables were expressed as medians and interquartile ranges (IQRs) or in absolute numbers and percentages (%), where appropriate. Categorial variables were compared with the chi-squared (χ²) test. Continuous variables were compared with Wilcoxon rank sum test.

We divided the patients in two groups according to their handgrip strength upon admission (adequate and reduced). A Kaplan-Meier survival curve was designed to estimate the time to death. Additionally, a Cox proportional hazards regression analysis was conducted to evaluate the relationship between handgrip strength and the hazard of the event occurring. Based on direct acyclic graphs, designed according to the previous literature on the topic, age and MPI classes were found to be potential confounders, and the model was adjusted accordingly. The results were expressed as hazard ratios (HR), obtained with the log-rank method. The validity of the models was assessed through the Gehan-Breslow method, and the analysis of hazard proportionality. The results were reported indicating *p*-values in reference to 95% confidence intervals (95%CI).

RStudio software (2024.09.0 + 375 version) was used for the statistical analysis.

## Results

We analyzed prospectively data from 322 elderly subjects with fragility hip fracture admitted to an Italian university orthopedic unit, of whom 242 (75.2%) were women. The median age was 84 years (IQR: 77–88). The median handgrip strength was 13 kg (12 kg among women, 18.5 among men), and 235 subjects (73.0%) were found to have a low handgrip strength, as described in Methods. Also, 205 subjects (63.7%) were found to have a low risk of adverse event, according to MPI, and 98 (30.4%) a moderate risk. Additionally, 85 subjects (26.6%) reported a history one or more previous fractures. All the patients underwent orthopedic surgery. The other characteristics of CGA and blood tests of the enrolled subjects are detailed in Table 1.

**Table 1:** Characteristics of the sample

Variable	Median	IQR
Age (years)	81	76–85
Handgrip strength (kg)	13	8–18
SPMSQ	2	0–4
GDS	4	2–5
BADL	5	4–6
IADL	5	2–8
Exton-Smith scale	14	11–17
MNA	23	21.5–26
CIRS	25	23–28
Hemoglobin (g/dl)	11.9	10.3–13.1
Serum creatinine (mg/dl)	0.98	0.84–1.25
Cholinesterase (U/l)	7560	6260–9109
Albumin (g/dl)	2.8	2.6–3.1
Vitamin B9 (ng/ml)	6.3	3.9–9.5
Vitamin B12 (ng/ml)	332	259–419
Vitamin D (ng/ml)	13.7	6.9–24.8
Parathormone (pg/ml)	67.2	51.0–94.4
Thyroid stimulating hormone (μU/l)	1.8	1.1–2.8
C-reactive protein (mg/l)	50.2	20.1–87.4
	n.	%
MPI– low risk	205	63.7
MPI– moderate risk	98	30.4
MPI– severe risk	19	5.9

BADL, Basic Activities of Daily Living; CIRS, Comorbidity Index Rating Scale; IADL, Instrumental Activities of Daily Living; IQR, interquartile range; MNA, Mini Nutritional Assessment; MPI, Multidimensional Prognostic Index; SPMSQ, Short Portable Mental Status Questionnaire

**Fig. 1:**
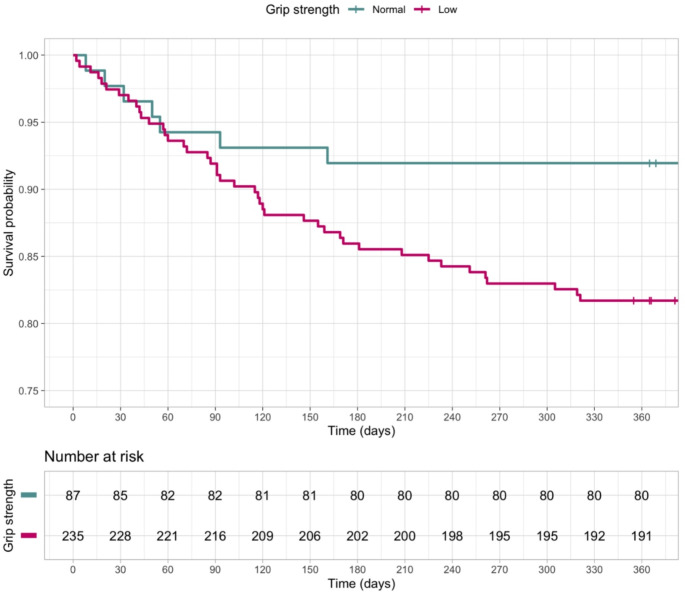
Kaplan-Meier survival curves stratified according to handgrip strength

The sample was divided according to handgrip strength in two subgroups: “low strength” (made up of the abovementioned 235 subjects), and “adequate strength” (87 subjects, 27.0%). Table 2 summarizes the differences in multidimensional domains between the two groups. In detail, all the assessed variables showed significantly more compromised scores in “low strength” group. After the one-year period, 50 people (15.5%) of the sample reached the outcome “death”. As displayed in Fig. 1, people belonging to “low strength” group reached the outcome significantly more frequently than people belonging to “adequate strength” group (*p* = 0.036). In detail, starting from two months after the fracture (60 days), the two survival curves significantly started to diverge. According to the Cox model, the HR for one-year death was 2.36 (95%CI: 1.06–5.24), as displayed in Table 3. We confirmed the validity of the model through the analysis of hazard proportionality (*p* = 0.076). According to the literature on the topic, we adjusted the model for potential confounders, namely age and MPI classes. The results showed that handgrip strength does not predict one-year mortality independently from age and MPI (HR: 1.31, 95%CI: 0.56–3.07), as displayed in Table 3. Nonetheless, the analysis of hazard proportionality showed a possible doubted validity of the adjusted model (*p* = 0.047).

**Table 2:** Comparison between “low strength” and “adequate strength” groups

Variable	Adequate strength	Low strength	*p*-value
	Median (IQR)	Median (IQR)	
Age (years)	79 (9)	85 (11)	< 0.0001
SPMSQ	1 (2)	2 (3)	< 0.0001
GDS	3 (3)	4 (3)	0.001
BADL	6 (1)	5 (2)	< 0.0001
IADL	8 (3)	4 (6)	< 0.0001
Exton-Smith scale	15 (7)	14 (5)	0.002
MNA	25 (4)	23 (5)	< 0.0001
CIRS	24 (4)	26 (6)	0.000
	n.	n.	
MPI– low risk	75	130	< 0.0001
MPI– moderate risk	10	88	
MPI– severe risk	2	17	

BADL, Basic Activities of Daily Living; CIRS, Comorbidity Index Rating Scale; IADL, Instrumental Activities of Daily Living; IQR, interquartile range; MNA, Mini Nutritional Assessment; MPI, Multidimensional Prognostic Index; SPMSQ, Short Portable Mental Status Questionnaire

**Table 3:** Summary of Cox regression models

Model	Adjusted for	Dependent variable	HR	95%CI
Unadjusted	-	Handgrip strength	2.36	1.06–5.24
Adjusted	Age and MPI scores	Handgrip strength	1.31	0.56–3.07

95%CI, 95% Confidence Interval; HR, Hazard Ratio; MPI, Multidimensional Prognostic Index

## Discussion

Fragility fractures in elderly people represent a significant public health issue [1]. As shown by several studies, age, comorbidities, reduced physical capacities, malnutrition, and reduced muscle strength can be considered as potential predictors of mortality in these patients. Among them, muscle strength, which is a crucial component of sarcopenia, has revealed a rapid, reproducible, and cost-effective assessment [5, 25].

This study was aimed at measuring the ability of handgrip strength to predict one-year mortality in elderly patients with fragility hip fracture. As for multidimensional tools, our sample showed overall adequate cognitive capacities and mood, and a low-to-moderate dependence in activities of daily living, as confirmed by the relatively low number of people with high risk of adverse event. Other studies with similar samples showed quite similar characteristics, with slightly more patients with higher severe risk of adverse event [39, 40]. This aspect can represent the place- and hospital- related variability, and it confirms the need of more studies on multidimensional assessment in orthogeriatric setting.

As for blood tests, albumin and vitamin D were overall found to be inadequate, consistently with numerous studies conducted on similar samples, as well as an inflammatory state, expressed by the high C-reactive protein values [41–44].

Handgrip strength was averagely low as well, and it was used as the categorical variable for our study. In fact, our sample was divided according to grip strength, following EWGSOP2 guidelines.

The one-year death rate was 15.5%. Several studies on fragility hip fractures examined this outcome and reported a wide range of results [1, 3, 4, 13, 39]. Generally speaking, it has been shown that collaborative management between orthopedic surgeons and geriatricians is related with lower mortality rates, whereas management solely by orthopedic surgeons seems to be related with higher one-year mortality rates [45–47]. Our sample aligns with this trend, as the one-year mortality that we found is lower than those reported by other studies. Nonetheless, we did not compare our data with that of the same unit before the introduction of orthogeriatric management, so we cannot determine the reasons for this finding.

Following the aim of our study, as previously stated, the patients were divided in two groups according to their handgrip strength (adequate or low). We noticed that, starting from day 60 (two months post-admission), the mortality rate was higher among people with lower baseline strength. Overall, we found that people with low handgrip strength have a 236% increased hazard for one-year mortality (*p* = 0.036). After adjusting for age and MPI, this ratio decreased to 131%, with a 95%CI crossing the line of no effect. However, the analysis of hazard proportionality invited us to be cautious before concluding that handgrip is not an independent predictor of mortality. As for the “delayed increase” in mortality among people with low handgrip strength, the 2-month mortality is not frequently explored in the scientific literature, but it could depend on the fact that the initial weeks following admission are usually marked by rehabilitation, frequently delivered in specialized facilities, and accompanied by close medical and nursing supervision. Upon the completion of this phase, the patients’ capacities to manage long-term complications become more evident, reflecting factors that transcend mere “physical stabilization”. This issue is also reflected in the higher overall compromission in several geriatric domains between the two groups analyzed in this study.

To the best of our knowledge, this study is one of the few to examine handgrip strength as a predictor of mortality in a such old population with hip fractures, and we believe it is the most significant strength of our work, even though the results deriving from the regression model were not significant. Several studies have explored handgrip strength as a potential predictor of mortality [48–50], but even reviews involving thousands of patients were not specifically devoted to elderly patients with hip fractures, a group who represents a key subject of study in medical research. Nonetheless, we acknowledge some limitations. Firstly, as previously discussed, a larger sample size would have clarified the influence of potential confounders without the risk of overfitting. Also, a more thorough assessment of sarcopenia, including an analysis of muscle quantity, according to EWGSOP2 guidelines, could have provided a more complete analysis than the handgrip strength alone. Finally, since this was a monocentric study, and two-thirds of the sample consisted of women, we cannot ensure the generalizability of our findings to the entire elderly population.

In conclusion, our study demonstrates that handgrip strength is a reliable predictor of one-year mortality in elderly people with fragility hip fracture. It also suggests that this association could be mediated by other variables, such as age and frailty, without allowing us to draw a definite conclusion. Further studies with larger samples are needed to clarify this issue. In any case, clinicians are warmly invited to perform handgrip measurement in elderly patients with hip fracture, due to the aforementioned reliability, together with its rapid and highly reproducible assessment.

## Data Availability

No datasets were generated or analysed during the current study.
